# Spatial Autocorrelation of Breast and Prostate Cancer in Slovakia

**DOI:** 10.3390/ijerph17124440

**Published:** 2020-06-20

**Authors:** Katarína Vilinová

**Affiliations:** Department of Geography and Regional Development, Faculty of Natural Sciences, Constantine the Philosopher University in Nitra, Tr. A. Hlinku 1, 94974 Nitra, Slovakia; kvilinova@ukf.sk

**Keywords:** medical geography, breast cancer, prostate cancer, spatial autocorrelation, Slovakia

## Abstract

Cancer is one of the dominant causes of death in the Slovak population. Monitoring the course of the cancer death rate in Slovakia can be considered as a relevant subject for geographical research. Relatively little is known about the geographic distribution of breast and prostate cancer incidence in Slovakia. In the submitted paper, it is hypothesized that breast and prostate cancer in the examined territory are characterized by different intensities, incidences, and spatial differences. The spatial patterns of breast and prostate cancer in Slovakia were examined by means of spatial autocorrelation analyses with the Local Moran’s I and Anselin Local Moran’s statistics. Data on standardized death rates of breast and prostate cancer in Slovakia between 2001 and 2018 were used. Prostate cancer in men and breast cancer in women show a positive statistically significant Global Moran’s I, whose values indicate a tendency to cluster. The Anselin Local Moran’s I analysis indicates significant clusters of breast cancer in the western part of Slovakia, and prostate cancer clusters mostly in the central part of Slovakia. The findings we have obtained in this study may help us investigate further hypotheses regarding the causes and identification of spatial differences in breast and prostate cancer incidence. Our findings might stimulate further research into the possible causes which underlie the clusters.

## 1. Introduction

In terms of death rates, the countries of the European Union are currently undergoing changes that are specific to individual countries. Their common trait in these terms is the dominant position of cancer mortality. Europe comprises only 9% of the world’s population, but as much as 25% of the global cancer burden. According to Reference [1], the estimates of the incidence and death rates at national level of European countries for 2018 are based on statistical models. The most recent published data have been used, with predictions derived from the most recent trends, where possible. The increase in some types of cancer (e.g., breast, colorectal, and lung cancer) is only one of the aspects that characterizes this condition. In analysing this phenomenon, we consider spatial differences to be very important, both in European countries and in other regions of the world [2,3]. A similar situation in mortality due to cancer is also observed in Slovakia. After the stabilization of mortality and morbidity from infection diseases, thanks to the implementation of the National Cardiovascular and Oncology Program in the 1980s, circulatory system diseases and cancer have become primary social interest [4]. Cancer mortality in the Slovak population is influenced by a large number of risk factors. An individual cancer is associated with various risk factors.

This fact is also characterized by statistics of cancer deaths in Slovakia. In 2001, the total mortality from cancer was 11,870 cases, which represents 22% of the total number of deaths. By 2018, the total mortality from malignancies had increased to 13,870 deaths (26% of the total deaths). It is very important to analyze cancer mortality from a gender perspective. In the case of both sexes, there is an increase in the number of deaths from this disease. The total number of women who died of cancer in Slovakia in 2001 was 4845 (18% of the total number of women who died). By 2018, the number of women who died of cancer had risen to 6113 (23% of the total number of women who died). A similar situation occurs with male cancer mortality. The number of men who died of cancer in 2001 reached 7025 (24% of the total number of men who died). The number of men who died of cancer increased to 7765 by 2018 (28% of the total number of men who died). In 2001, the standardized death rate of cancer of men was 316.5/100,000 inhabitants and women 152.6/100,000 inhabitants. The standardized death rate of cancer of men in Slovakia in 2018 was 259.8/100,000 inhabitants and women 142.9/100,000 inhabitants.

Breast cancer is the most frequently diagnosed cancer worldwide, which is also the most common cause of cancer death in women in Slovakia [5]. The factors that most influence breast cancer, both in Slovakia and the rest of the world, include age and gender, lifestyle, reproductive and hormonal factors, family history, genetic, environmental factors, smoking, and others. Prostate cancer is the second most common cause of cancer mortality in men in Slovakia. In the case of this mortality, lifestyle, socioeconomic status, income, and access to healthcare are considered risk factors.

The study of spatial disparities in cancer incidence is a relatively new area of interest in Slovakia, focusing on their specificities, as well as the assessment of their risk factors. The issue of mortality, as one of the main demographical processes, is also addressed in geographical research works. [6,7,8,9,10]. However, in Slovakia, such research efforts used to lag behind in the past. The issue of mapping in a detailed spatial resolution has been addressed by Reference [11], who have intorduced the most frequently applied models in spatial epidemiology. Although socioeconomic factors are not directly linked to the development of cancer, they create conditions leading to a higher incidence of risk factors for carcinogenesis. These risk factors include, for example, smoking, alcohol consumption, unhealthy eating habits, lack of physical activity, exposure to carcinogens, and access to healthcare (such as screening and treatment), which are directly linked to mortality and survival [12].

The reasons why some people develop a cancer and others do not are unknown. National oncological registries and the implementation of the “Europe Against Cancer” programme play an important role in monitoring, planning, and evaluating national plans. The European Society of Breast Imaging (EUSOBI), for example, is a major contributor to prevention. It is dedicated to promoting research and education on the best screening, diagnosis, and intervention measures, for which mamographs and screening are the main tasks. They are considered important for reducing mortality and breast cancer treatment, based on an example comparison of 30 countries in Reference [13]. In prostate cancer, as in breast cancer, screening measures are being introduced [14]. The aim of the study is to analyze and evaluate mortality due to selected types of cancer, namely breast and prostate, in the period from 2001 to 2018. The observed unit of the study is the Slovak Republic. The analyses were conducted on the district level. The phenomena evaluation is characterized by both the time and spatial aspect. Moreover, our objective is to identify regions based on a homogeneity criterion. Mortality is assessed by means of the standardized death rate of breast cancer and the standardized death rate of prostate cancer. The growing trend of these two types of cancer in Slovakia (breast cancer and prostate cancer) is the main problem examined for us in the study. The absence of elaboration of this issue in the Slovak literature, both in time and space, led us to take an interest in the elaboration of the issue.

## 2. Materials and Methods

Judging by the very nature of the available data, as well as the applied analytical approaches, from the methodological point of view, this article may be regarded as a retrospective cross-sectional analysis. These are typical of space-oriented and epidemiological research practice. Death rate is one of the demographic indicators which is the most common for the use of standardization. The crude death rate depends on the intensity of mortality in each age group and on the age structure of the population. Obviously, this indicator is not suitable for comparison if the populations have a different age structure. On the other hand, in order to make the result of the comparison meaningful, it is sufficient to eliminate the influence of the age structure.

For the purposes of the analysis, calculated annual standardized death rates of breast cancer and prostate cancer were used by means of direct standardization, where the age-specific death rates of the real population are applied to the standard population. In this study, one of the most commonly used, the European Standard Population, was applied to assess mortality.

The calculation provided us with the death rate that would occur in the real population if its age structure corresponded to the age structure of the standard population. In further sections, the standardized death rate is calculated per 1000 inhabitants of the European Standard Population. This analytical view was applied separately for three 6-year periods (2001–2006, 2007–2012, and 2013–2018) for the standardized death rate of breast cancer and for the standardized death rate of prostate cancer.

The technique of spatial autocorrelation is often used in scientific research. Already in the 1940s, Cruickshack pointed to the presence of positive autocorrelations in relative cancer mortality rate in England and Wales [15]. Identification of clusters of districts by statistically higher and lower levels of mortality provided the opportunity for research to focus on these areas. At the same time, the objective was to detect the effects of local environmental factors on the higher or lower death rates of breast and prostate cancer. That is why spatial analyses of cancer data are currently very often used in practice [16]. According to Reference [17], spatial analyses are mainly used to uncover the relationship between geography and health. One such method is the method of spatial correlations. For example, References [18,19] apply this method to breast cancer, while Reference [20] applies it to prostate cancer.

In this study, we apply the method of spatial autocorrelation in order to evaluate the standardized death rate of breast cancer in women and the standardized death rate of prostate cancer in men in Slovakia. The analysis is performed in relation to three periods (2001–2006, 2007–2012, and 2013–2018). Relative rates assessed in a space–time context are used for the purpose of evaluating the prevalence of the researched subject and its subsequent comparison with other geographical entities. The main bases of the processed data were the data provided by the Statistical Office of the Slovak Republic, namely the DATAcube database (section Demography and Social Statistics—Population—Demographical Processes—Deaths; spreadsheets Deceased by Death Causes, Gender and Address).

A spatial cluster analysis of breast and prostate cancer cases was performed, using spatial autocorrelation. Spatial data analysis was performed by using the free software GeoDa. A spatial cluster model of the overall area was estimated via the global spatial autocorrelation index Moran’s *I*. In the context of the methods applied, Moran’s index and Geary’s coefficient are commonly used for continuous data analysis. The effectiveness of Moran’s index is generally slightly better than Geary’s coefficient [21].

Moran’s index is defined as follows:

(1) $$I=\frac{n\left(\sum_{i=1}^{n}\sum_{j=1}^{n}w_{ij}\left(x_{i}-\bar{x}\right)\left(x_{j}-\bar{x}\right)\right)}{\left(\sum_{i=1}^{n}\sum_{j=1}^{n}w_{ij}\right)\left(\sum_{i=1}^{n}{\left(x_{i}-\bar{x}\right)}^{2}\right)}$$

where *n* is the number of spatial units (in this case it is the number of all district of Slovakia); *x_i_* is the value of the variable in the region *i* (standardized death rate of breast cancer per 1000 inhabitants European Standard Population, standardized death rate of prostate cancer per 1000 inhabitants European Standard Population); *x* is the arithmetic average for the given variables; and *w_ij_* is the spatial weight.

Moran’s index was used to measure the spatial autocorrelation. Values of Moran’s index (*I*) range from −1 to +1. The further away it is from zero, the stronger (positive or negative) the autocorrelation. When *I* > 0, the disease distribution is positive for spatial autocorrelation and vice versa. A positive autocorrelation means that values in one area are similar to those in neighboring areas, whereas a negative autocorrelation means that, if one area has a high incidence rate, the neighborhood areas have low incidence rates.

Local Moran’s index, known as the local index of spatial autocorrelation (LISA), was used. LISA analysis allowed the identification of the existence of spatial clusters or regions with high or low values for the analyzed variables. There can be five different scenarios within the LISA: (1) high-high, “hot spots”; (2) low-low, “cold spots”; (3) high-low, “spatial outliers”; (4) low-high, again “spatial outliers”; and (5) no significant local spatial autocorrelation. This methodology allows the identification of regions that contribute to spatial autocorrelation.

It is important to note that the size of Moran’s index itself does not indicate the statistical significance. Statistical significance of the calculated values, which rejects the null hypothesis of non-existence of spatial autocorrelation, was verified by the permutation method, using the software GeoDa, when all values are considered statistically significant at 1% level of significance.

For the analysis of the detection of specific spatial clusters, the local version of the Moran I criterion [22] was used, which evaluates the degree of autocorrelation of a spatial quantity between a given point of the space and its surroundings. The corresponding indicator is suitable for the location of units with relatively high (i.e., above-average) or low (i.e., below-average) values (so-called positive spatial correlation) or sudden turns in spatial distribution of the studied phenomena (negative spatial correlation). The statistical interference of all three applied indicators (General G, Moran’s I criterion, and Local Moran’s I) is based on the calculation of the Z-score.

Moran’s I: Local Moran´s

(2) $$\begin{array}{l} I=\frac{N}{\sum_{i}^{N}\sum_{j}^{N}w_{i,j}}\frac{\sum_{i}^{N}\sum_{j}^{N}w_{i,j}\left(X_{i}-\bar{X}\right)\left(X_{j}-\bar{X}\right)}{{\left(X_{i}-\bar{X}\right)}^{2}}\;I_{i}=\frac{X_{i}-\bar{X}}{S_{i}^{2}}\overset{N}{\underset{j}{\sum }}w_{i,j\;}\left(X_{ij}-\bar{X}\right)\\ i,\;j=1,\;\ldots ,\;N=57;\;i\neq j\;S_{i}^{2}=\frac{\sum_{j}^{N}{\left(X_{j}-\bar{X}\right)}^{2}}{N-1}-{\bar{X}}^{2}\\ i,\;j\;1,\;\ldots ,\;N=57;\;i\neq j \end{array}$$

Getis-Ord

(3) $$\begin{array}{c} G=\frac{\sum_{i}^{N}\sum_{j}^{N}w_{i,j}X_{i}X_{j}}{\sum_{i}^{N}\sum_{j}^{N}X_{i}X_{j}}\\ i,\;j=1,\;\ldots ,\;N=57;\;i\neq j \end{array}$$

## 3. Results

The beginning of the 1990s brought a new era of death rates in Slovakia. Its main feature was a more or less continuous reduction in the overall death rate and the prolongation of life [23]. There were a number of interacting factors which allowed the improvement of death rates and overall health condition of the Slovak population after 1989. First of all, it was the improvement in healthcare as a result of the growing volume of healthcare funding. Another positive impulse was opening the market, which brought the possibility to exchange experience; the access to the latest medical techniques, methods, and procedures; and also the availability of a variety of modern medications and equipment. However, in the following period, various trends in death rates were accompanied by re-widening differences. The main driving forces behind the positive developments in death rates in the western block were, in particular, the reduction of mortality from cardiovascular diseases and some forms of cancer, and also the prevention of illnesses associated with certain behavioral factors.

**Figure 1:**
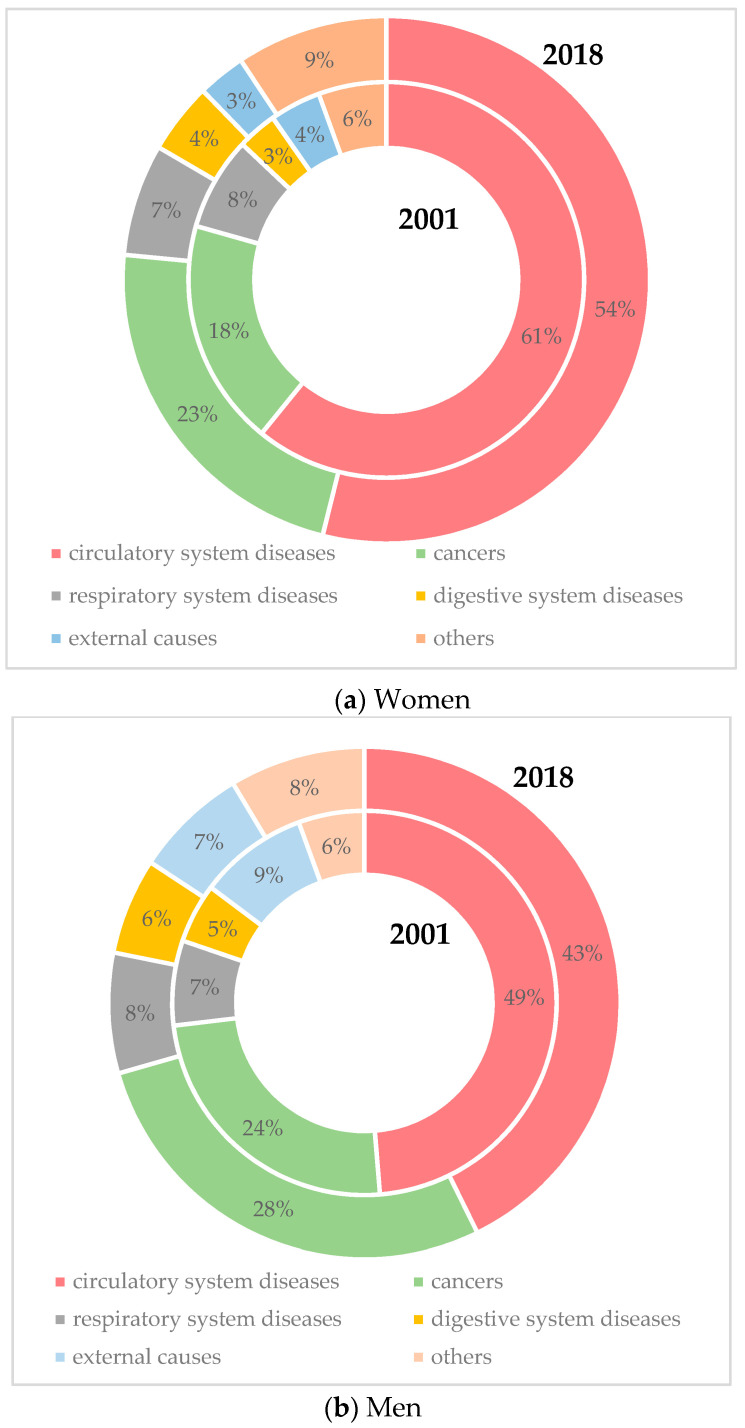
Population mortality by causes of death. Source: Statistical Office of the Slovak Republic, 2019.

An important aspect while evaluating the course of mortality is the study of death causes. Figure 1 summarizes the structure of death causes in men and women in Slovakia in 2001 and 2018. It has long been known that the highest death rate is recorded for circulatory system diseases. This cause dominates the mortality of men but also of women. However, the death rate for this cause is higher in women. Another aspect that we see is the decline in mortality from this cause in both sexes. The decline of the death rate was 7% in women and 6% in men in the analyzed years. Cancer was the next most common death cause in the Slovak population. The Slovak population’s share of the overall mortality in 2018 was 23% in women and 28% in men. This death cause is characterized by an increase when comparing 2001 and 2018. Another three most common death causes are respiratory diseases, digestive diseases, and exogenous causes. When comparing the cancer death rate of Slovaks with other EU countries, different characteristics from that of the circulatory mortality can be observed. In female cancer mortality, the Slovak Republic is one of the countries with an average death rate. However, this cannot be stated about Slovak men, since regarding the male cancer death rate, Slovakia belongs to countries with the highest mortality, although the difference is not so distinct.

Cancer is the second most common cause of death cause in Slovakia. This structure is dominated by mortality from breast cancer. In 2001, the standardized death rate of breast cancer in Slovakia was 28.9/100,000 inhabitants. By 2018, the standardized rate of breast cancer deaths increased; the standardized death rate of breast cancer in Slovakia in 2018 was 38.9/100,000 inhabitants. It is more than the standardized death rate of breast cancer in EU in 2018 (32.8/100,000 inahabitans).

The second most common type of cancer in male mortality was prostate cancer. From 2001 to 2018, the standardized rate of prostate cancer for men increased. In 2001, the standardized death rate of prostate cancer in Slovakia was 45.1/100,000 inhabitants. The standardized death rate of prostate cancer in Slovakia in 2018 was 50.7/100,000 inhabitants. It is more than the standardized death rate of breast cancer in EU in 2018 (39.1/100,000 inahabitants).

### 3.1. Spatial Specifics of Breast Cancer in Slovakia

#### 3.1.1. Spatial Autocorelation of Breast Cancer in Slovakia

**Figure 2:**
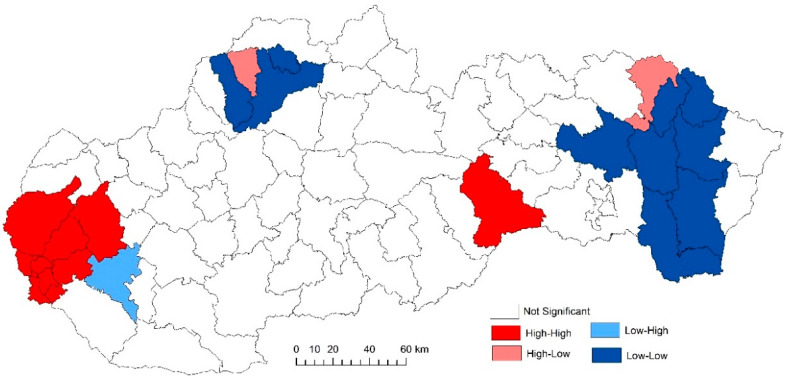
Reginalization of Slovakia based on the local index of spatial autocorrelation (LISA) analysis for standardized death rate of breast cancer per 1000 inhabitants European Standard Population, 2001–2006. Source: Statistical Office of the Slovak Republic, 2019.

**Figure 3:**
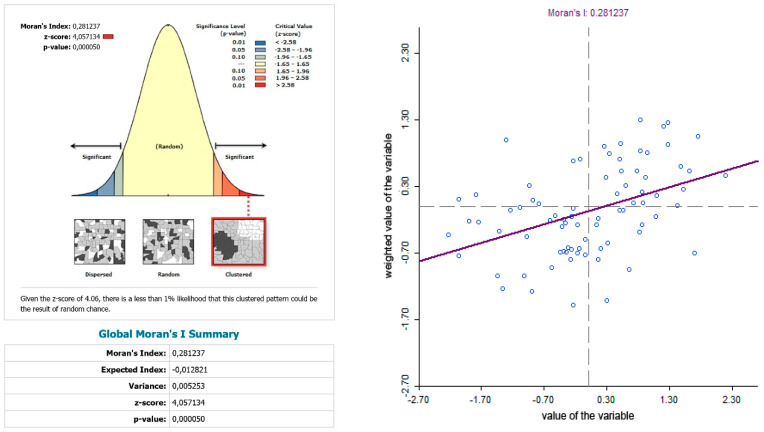
Final statistics and Moran′s diagram for standardized death rate of breast cancer per 1000 inhabitants of European Standard Population 2001–2006. Source: Statistical Office of the Slovak Republic, 2019.

**Figure 4:**
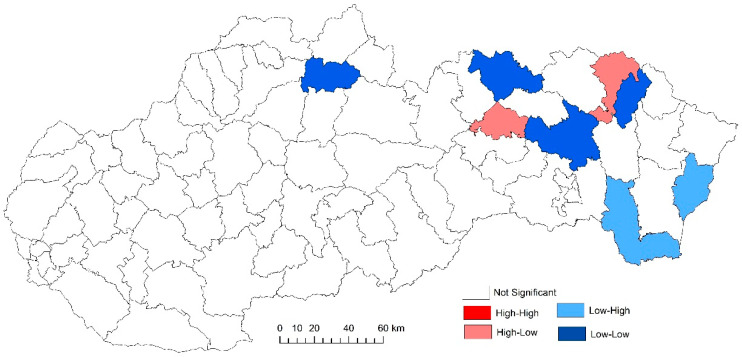
Reginalization of Slovakia based on the LISA analysis for standardized death rate of breast cancer per 1000 inhabitants of European Standard Population, 2007–2012. Source: Statistical Office of the Slovak Republic, 2019.

**Figure 5:**
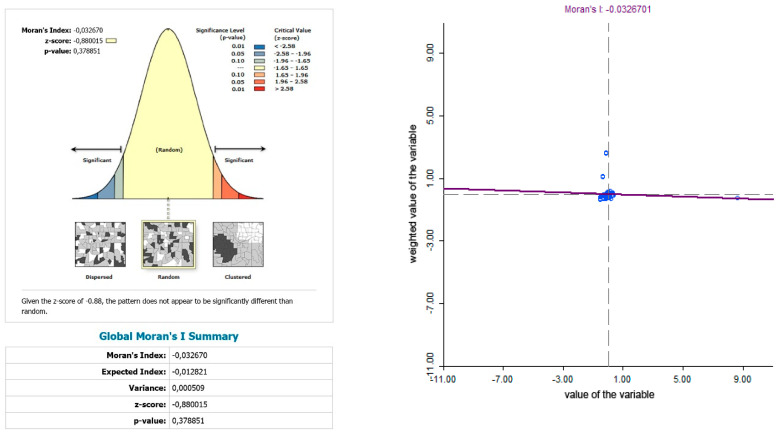
Final statistics and Moran′s diagram for standardized death rate of breast cancer per 1000 inhabitants of European Standard Population, 2007–2012. Source: Statistical Office of the Slovak Republic, 2019.

**Figure 6:**
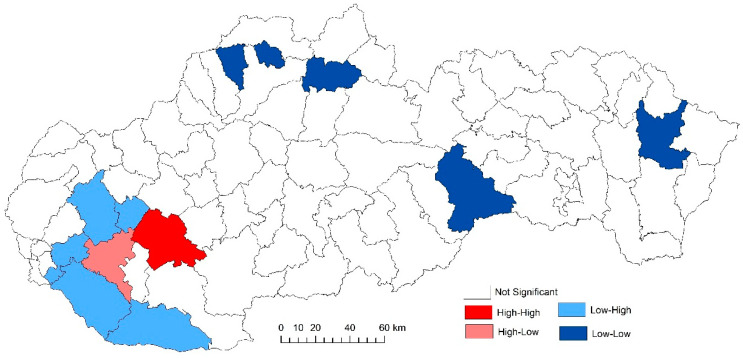
Reginalization of Slovakia based on the LISA analysis for standardized death rate of breast cancer per 1000 inhabitants of European Standard Population, 2013–2018. Source: Statistical Office of the Slovak Republic, 2019.

**Figure 7:**
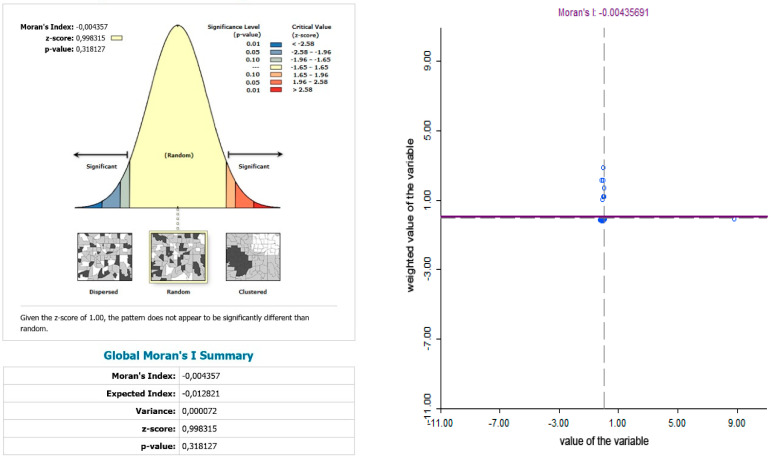
Final statistics and Moran′s diagram for standardized death rate of breast cancer per 1000 inhabitants of European Standard Population, 2013–2018. Source: Statistical Office of the Slovak Republic, 2019.

The use of spatially referenced data on standardized death rates for breast and prostate cancer in Slovak districts provides us with an understanding of the spatial-analytical links in this territory. It is by means of the spatial correlation method that it is possible to identify the clusters of regions with respect to the monitored indicators. Having assessed the spatial autocorrelation of breast cancer, sites with varying intensity of incidence of breast cancer standardized death rate were identified in the periods 2001–2006, 2007–2012, and 2013–2018. This can be observed as positive or negative spatial autocorrelation. When analysing the standardized death rate of breast cancer in the period 2001–2006, such districts can be identified, especially in the western part of Slovakia, with the district of Rožňava, which is found in the high-high quadrant (Figure 2). The Moran’s diagram for the indicator of breast cancer standardized death rate in 2001–2006 assumed the 0.281237, which implies a moderate positive spatial autocorrelation (Figure 3). This value indicates clustering of close values of the breast cancer standardized death rate (high with high; low with low). As already mentioned, the Global Moran’s index on its own does not reveal different levels of spatial links within one dataset. Other examples are the districts in the eastern part of Slovakia (Stropkov, Medzilaborce, Prešov, Vranov nad Topľou, Humenné, Trebišov, and Michalovce) and the districts of Považská Bystrica, Žilina, and Kysucké Nové Mesto, whose breast cancer standardized death rates are found in the low-low quandrant. In the following period, i.e., 2007–2012, the situation changed with respect to the examined indicator. Four Slovak districts (Dolný Kubín, Stará Ľubovňa, Prešov, and Stropkov) were found in the low-low quadrant (Figure 4). The Moran’s diagram for 2007–2012 shows that the examined indicator assumed value −0.0326701, which, in global measures, implies a negative spatial autocorrelation (Figure 5). Significant changes in relation to the spatial autocorrelation of the breast cancer standardized death rate also took place in 2013–2018. It can be stated that, based on the spatial autocorrelation of this period, only one district in the west of Slovakia (Nitra) was found in the high-high quadrant. In the low-low quadrant, several districts were isolated, namely Bytča, Kysucké Nové Mesto, Dolný Kubín, Rožňava, and Humenné (Figure 6). The index of the Moran’s diagram in this period assumed value −0.00435691, which implied a negative spatial autocorrelation (Figure 7). The causes of spatial disparities of this phenomenon are closely related to several factors, which, however, are difficult to identify. Since neoplastic diseases are multifaceted, it is demanding to specify the risk factors for the inception of these diseases. Any direct dependence on risk factors cannot be clearly specified. Despite this fact, the most important factors behind this disease are environmental quality, stress, lifestyle, age composition of the population, poverty rates, and others. From this perspective, it is not possible to express a direct dependence on the high values of standardized death rate and the level of regional development in Slovakia.

#### 3.1.2. Standardized Death Rate of Breast Cancer

**Figure 8:**
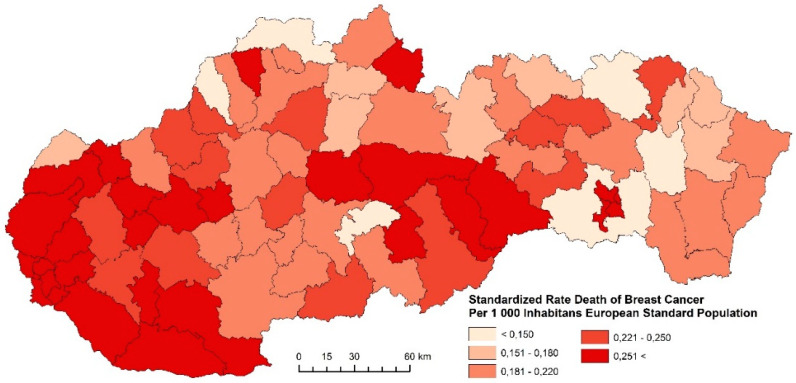
Standardized death rate of breast cancer per 1000 inhabitants of European Standard Population, 2001–2006. Source: Statistical Office of the Slovak Republic, 2019.

**Figure 9:**
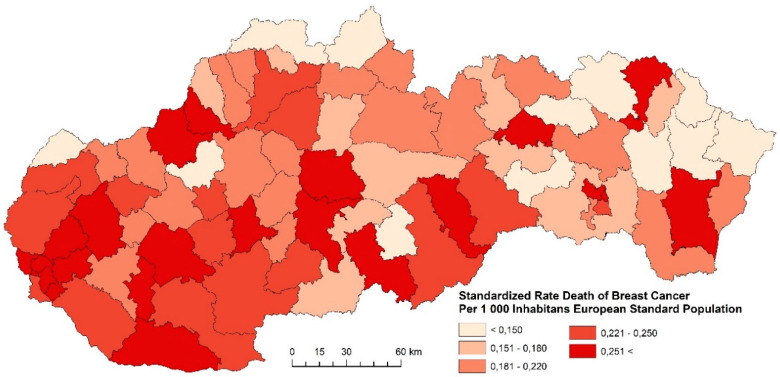
Standardized death rate of breast cancer per 1000 inhabitants of European Standard Population, 2007–2012. Source: Statistical Office of the Slovak Republic, 2019.

**Figure 10:**
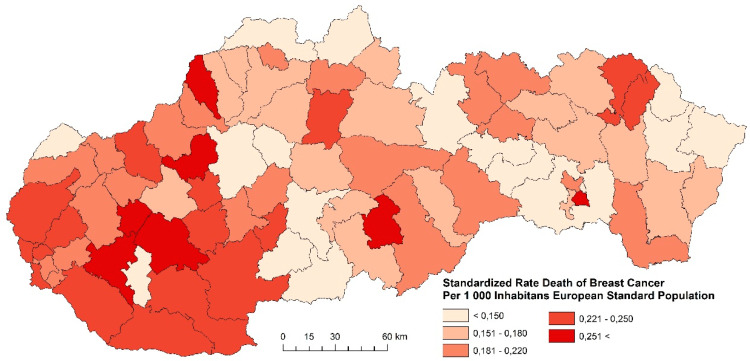
Standardized death rate of Breast Cancer per 1000 inhabitants of European Standard Population, 2013–2018. Source: Statistical Office of the Slovak Republic, 2019.

The standardized death rate of breast cancer in the examined periods, with respect to regional differences, is summarized in Figure 8, Figure 9 and Figure 10. The indicator of breast cancer standardized death rate in Slovakia indicates significant spatial differences. The differences are related to the period whose indicator is studied. The period 2001–2006 is characterized by homogeneous regions with districts whose indicator assumes the highest values more than 0.251 and more persons per 1000 inhabitants of the European Standard Population. These regions were identified mainly in the western and central parts of Slovakia (Figure 8). Compared to the previous period, the period 2007–2012 is characterized by a decrease of the indicator in most Slovak districts. Very favorable results were identified in the eastern and northern districts (Figure 9). Judging by the results in the last examined period, in 2013–2018, the breast cancer standardized death rate was successfully decreased, as shown in Figure 10. As few as six districts (Nitra, Hlohovec, Galanta, Bánovce nad Bebravou, Púchov, Poltár, and Košice IV) reached the values of breast cancer standardized death rates higher than 0.251 and more persons per 1000 inhabitants of the European Standard Population. We suppose that the reason for the decrease in breast cancer mortality may be, e.g., screening. This leads to an increase in the number of registered cancer cases with a smaller extent of the disease. Diagnosing them at an earlier stage leads to a reduction in mortality and improved survival [5]. Another group of factors includes improvements in the management of disease treatment, including chemotherapy and hormonal treatment, by reorganizing healthcare, which includes a multidisciplinary approach.

### 3.2. Spatial Specifics of Prostate Cancer in Slovakia

#### 3.2.1. Spatial Autocorelation of Prostate Cancer in Slovakia

**Figure 11:**
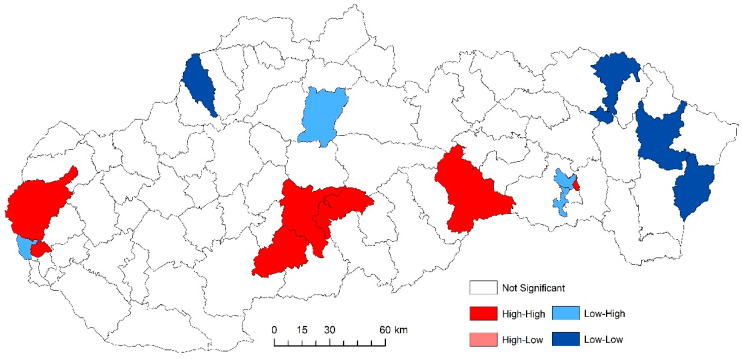
Reginalization of Slovakia based on the LISA analysis for standardized death rate of prostate cancer per 1000 inhabitants of European Standard Population, 2001–2006. Source: Statistical Office of the Slovak Republic, 2019.

**Figure 12:**
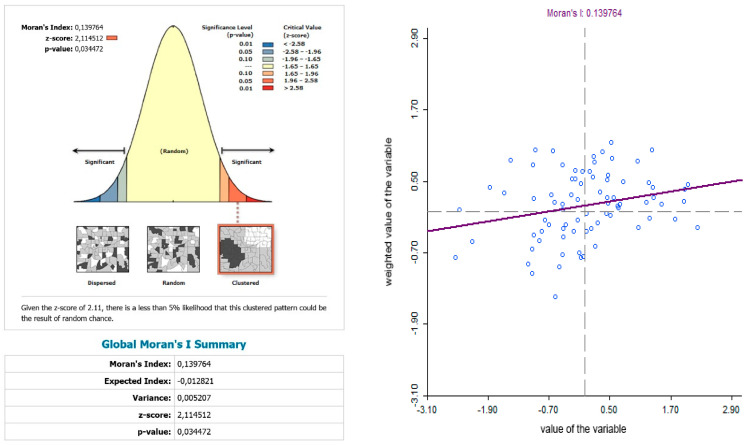
Final statistics and Moran′s diagram for standardized death rate of breast cancer per 1000 inhabitants of European Standard Population, 2001–2006. Source: Statistical Office of the Slovak Republic, 2019.

**Figure 13:**
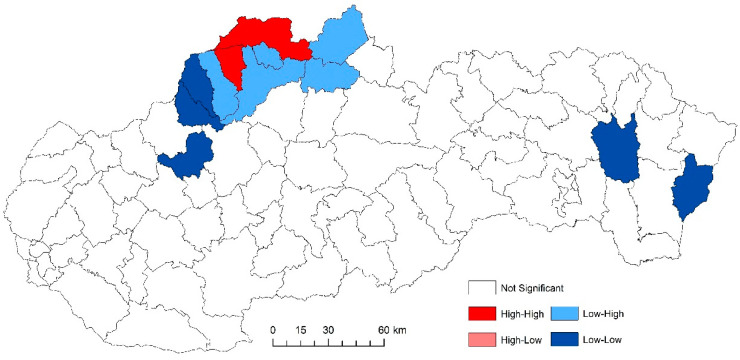
Standardized death rate of prostate cancer per 1000 inhabitants in European Standard Population, 2007–2012. Source: Statistical Office of the Slovak Republic, 2019.

**Figure 14:**
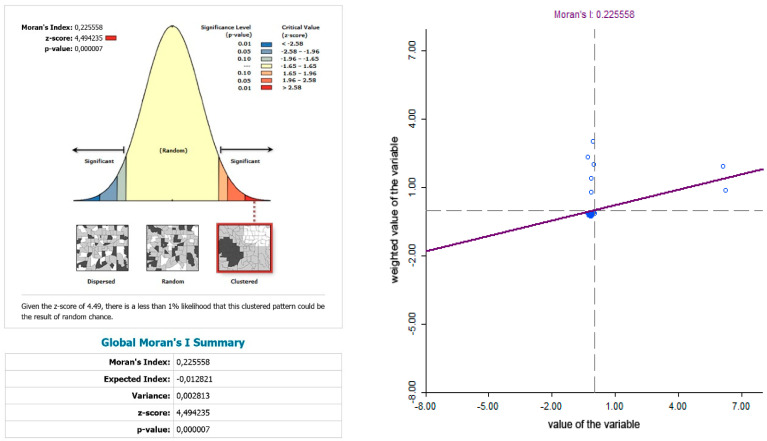
Standardized death rate of prostate cancer per 1000 inhabitants in European Standard Population, 2007–2012. Source: Statistical Office of the Slovak Republic, 2019.

**Figure 15:**
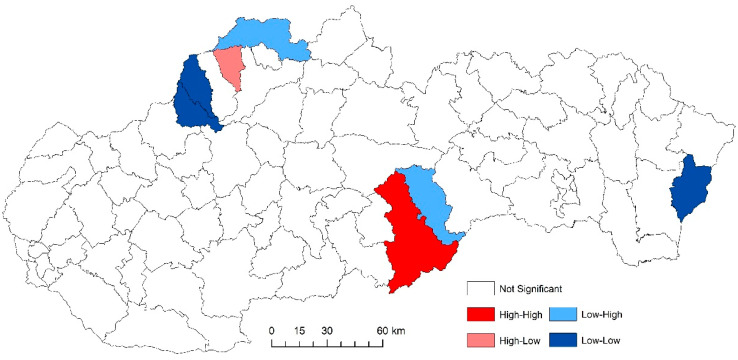
Standardized death rate of prostate cancer per 1000 inhabitants of European Standard Population, 2013–2018.

**Figure 16:**
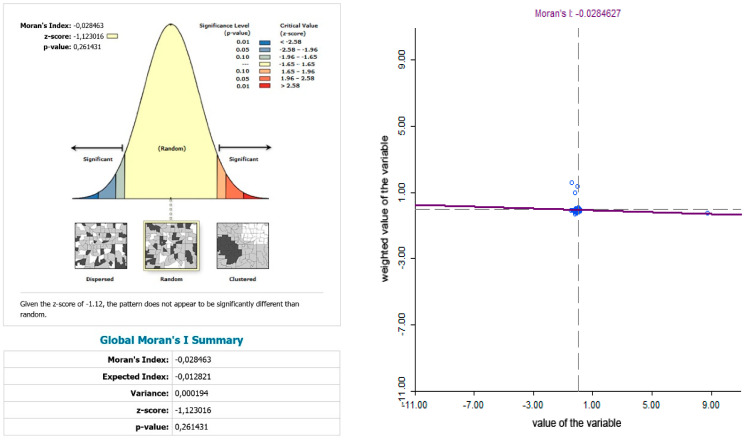
Standardized death rate of prostate cancer per 1000 inhabitants of European Standard Population, 2013–2018. Source: Statistical Office of the Slovak Republic, 2019.

While examining the spatial incidence of the prostate cancer standardized death rates, the Local Moran’s I, with respect to Slovak districts in the studied territory and time periods, indicates both positive and negative spatial autocorrelation. According to Reference [24], a positive spatial autocorrelation means that geographically close values of the variable (neoplastic disease standardized death rate) tend to cluster with similar values of the variable in a map, i.e., high values tend to be located close to high values, medium values tend to be located close to medium values, and low values are located close to low values. In period the 2001–2006, both positive and negative spatial autocorrelation can be observed (Figure 11). The first “high-high” cluster in this period is located in seven Slovak districts, and out of them, three districts (Krupina, Zvolen, and Detva) comprise one compact region. The other four “high-high” districts are Rožňava, Košice III, Malacky, and Bratislava III. In this period, the index of the indicator from the Moran’s diagram assumed value 0.139764 (Figure 12). In the low-low quandrant, four districts are found, namely Púchov, Svidník, Humenné, and Sobrance. As the previous analysis has shown, it can be stated that, in the 2007–2012 period, positive, as well as negative, spatial autocorrelation is observed. The index value has risen to 0.225558 (Figure 13). Most of the clusters are located in the north of Slovakia. The high-high cluster comprises thedistricts Bytča and Čadca. Districts located in various parts of Slovakia (Sobrance, Vranov nad Topľou, Bánovce nad Bebravou, Púchov, and Ilava) are characterized by positive spatial autocorrelation in the low-low quandrant (Figure 14). Similarly, in period the 2013–2018, both positive and negative spatial autocorrelations of the examined phenomenon have been identified, while only one district, namely Rožňava, is in the high-high category (Figure 15). The low-low quadrant includes the districts Sobrance, Púchov, and Ilava. In this period, the maximum value of the Moran’s I was −0.0284627 (Figure 16), which is also a case of negative spatial autocorrelation.

#### 3.2.2. Standardized Death Rate of Prostate Cancer

**Figure 17:**
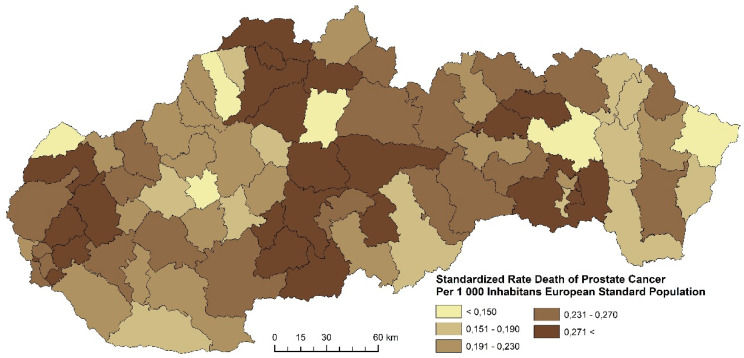
Standardized death rate of prostate cancer per 1000 inhabitants of European Standard Population, 2001–2006. Source: Statistical Office of the Slovak Republic, 2019.

**Figure 18:**
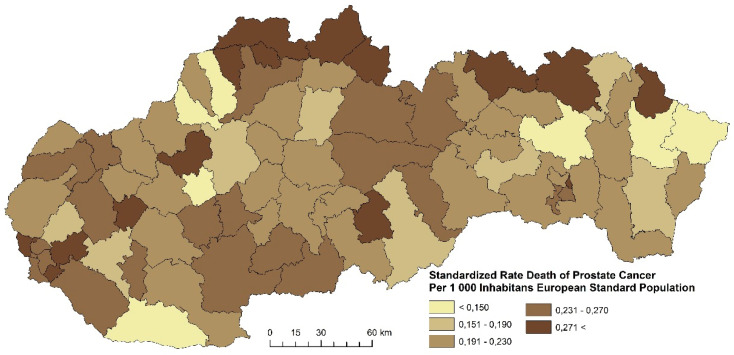
Standardized death rate of prostate cancer per 1000 inhabitants of European Standard Population, 2007–2012. Source: Statistical Office of the Slovak Republic, 2019.

**Figure 19:**
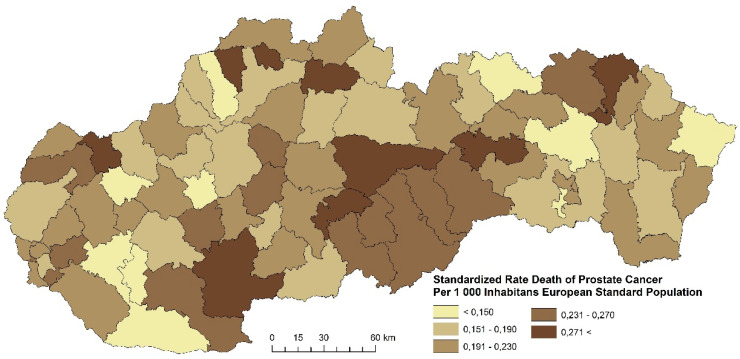
Standardized death rate of prostate cancer per 1000 inhabitants of European Standard Population, 2013–2018. Source: Statistical Office of the Slovak Republic, 2019.

The prostate cancer standardized death rate in men in Slovakia is characterized by a varied intensity, as well as spatial disparities. The distribution of the standardized death rate values varies in Slovakia. In the period 2001–2006, four regions were formed with the highest prostate cancer death rate (more than 0.271 per 1000 inhabitants of the European Standard Population). Altogether, 23 districts were identified in this category (Figure 17). The following examined period is characterized by the fact that most districts with the highest death rates are located in the north of Slovakia (Figure 18). This group includes districts Košice III, Poltár, Bánovce nad Bebravou, Hlohovec, Senec, and Bratislava IV. Figure 19 summarizes the prostate cancer standardized death rates in the period 2013–2018. This period is characterized by an overall decrease of the standardized death rate in most Slovak districts.

## 4. Discussion

Breast cancer retains a dominant position in the mortality of women. Breast cancer is the main cause of female cancers in Europe. It is estimated to affect more than one in ten women and represents 28.8% of female cancers. Breast cancer is by far the most commonly diagnosed malignancy, which is also the most common cause of death in women with an oncological disease.

Recently, especially in the foreign literature, research on these issues has moved from national and regional to rather intraregional and local levels. Research into the spatial disparities of the breast cancer death rate is relatively well-developed in the foreign literature. Most of these studies are focused on monitoring the breast cancer death rate from the perspective of spatial disparities [25,26,27,28]. Besides the spatial aspect, there are other factors underlying the disease to some extent. Education is an important socioeconomic factor that affects breast cancer death rate. A cohort study by Reference [29] has shown that the breast cancer death rate is higher in Norwegian women who received higher education in comparison to women with a lower education. The positive association between education and breast cancer death rate in women has also been indicated in studies from the USA [30], Belgium [31], and also the recent international study by Reference [32]. Individual factors, e.g., ethnic origin, family anamnesis, age, reproductive factors, alcohol consumption, weight, physical activity, and hormone treatment, have a significant influence on the risk of developing breast cancer. Another specificity is that we see different approaches to diagnosis, access to healthcare, and BMI values, but also different levels of stress burden, or social aspects that have an impact on the development of this disease [33]. Cancer epidemiology increasingly recognizes the impact of socioeconomic status (SES) on the incidence and progression. A high SES is associated with a higher risk of breast cancer and malignant melanoma, while a low SES is associated with an adverse progression, for example, in the case of stomach cancer, lung cancer, prostate cancer, and ovarian cancer [34,35].

A considerable amount of resources in the literature address issues relating to breast cancer by incidence, diagnosis, treatment, survivorship, survival, and mortality [36]. However, there is growing evidence of socioeconomic status (SES) gradients along the stages of the breast cancer continuum [37,38]. Several studies have reported a positive relationship between area level SES and incidence of breast cancer [39,40,41].

Prostate cancer is one of the most common malignancies in men in Europe [42,43]. It is the second most common cancer in men in the world and is even the first in Europe. Compared to Asia, which is known as the region with the lowest incidence of prostate cancer, it has been increasing significantly over the last 20 years [44]. One important measure that leads to a significant reduction in mortality from prostate cancer in the world but also in Europe is prostate cancer screening [14]. Differences in death rates between EU countries are the result of several factors. These include, for example, early detection (largely due to screening programmes, but also due to awareness-raising) of neoplastic diseases, and gradual improvement in treatment [45]. A similar situation in mortality from prostate cancer can be seen in Slovakia. In men, this is the second leading cause of death among men with cancer. According to worldwide estimates, Slovakia is classified as a country with a medium–high prostate cancer incidence. Current predictions indicate that, in the near future, prostate cancer will become the most common cancer among men in Slovakia [46]. A more thorough analysis of the problem would be needed to draw more general conclusions on possible causes, as is the case, for example, with prostate cancer research in relation to agriculture [47,48], the accessibility of health facilities [49], etc. [50].

Socioeconomic factors influence the risk of prostate cancer. Prostate cancer incidence rates tend to be positively associated with socioeconomic status. On the other hand, low socioeconomic status is associated with increased risk of poorer survival [51]. Social determinants of health that have been examined in relation to prostate cancer incidence, stage at diagnosis, and survival include socioeconomic status (income and education), immigration status, social support, and social network [52,53,54,55].

Breast and prostate cancer are analyzed together in some scholarly articles. An example is the study of a correlation between breast cancer and prostate cancer in men in the United States, between 2000 and 2005. Their results indicate that breast and prostate cancers are spatially highly co-clustered. This is, for example, in line with the results of other studies [56,57,58] that have identified comparative risk factors for these two cancers. Breast cancer and prostate cancer are the two most common invasive cancers in women and men, respectively. Although these cancers arise in organs that are different in terms of anatomy and physiological function, both organs require gonadal steroids for their development, and cancers that arise from them are typically hormone-dependent and have remarkable underlying biological similarities [59].

In order to investigate, analyze, and explain spatial patterns of breast cancer and prostate cancer in Slovakia, we have applied the techniques of spatial autocorrelation, both global and local methods. The results show that in significant clusters of incidence rate in various regions of the country there are rather large geographical disparities. The results showed that there is a relatively large geographical difference in significant clusters of the incidence rates between the areas of the country. The results of the local indices show the existence of significant clusters in the western and central regions of the country, which are likely to indicate the presence of common predisposing factors in these regions. Social factors (unemployment, poverty rates, marginalized groups, and others) are dispersed rather unevenly in the territory of Slovakia, which is reflected in the level of their socioeconomic development, which highly varies in regions. In some districts, this may be related to higher levels of standardized death rates of breast and prostate cancer. Among other things, some districts in Slovakia (e.g., Spišská Nová Ves, Levice, Rimavská Sobota, Bardejov, and others) belong to districts that are burdened with so-called old waste-disposal sites, as well as sites abandoned by their former mainly agricultural, but also industrial, plants that will have to be dismantled in order to avoid further pollution of the soil and groundwater, and, eventually, related health risks [60]. The map outputs imply a range of information, related to both content and spatial aspects of the regional differences in the standardized death rate of breast and prostate cancer. From the assessment of the results of the regional disparities of the breast cancer standardized death rate, it can be concluded that its dominance is predominantly located in the western part of Slovakia. The eastern part of Slovakia is significantly more favorable in terms of this indicator. In the case of prostate cancer standardized death rate, the situation is different in each studied period. In relation to prostate cancer death rate, the central, northern, and southern parts of Slovakia show unfavorable numbers. Despite the economic development of Western Slovakia, where a lower cancer death rate might have been assumed in association with a higher quality of life, as well as better healthcare, this fact has not been confirmed. Spatial disparities of neoplastic breast and prostate diseases described in this article reject the assumption. One possible factor that may have a significant impact on this is a greater influence of stress, more industrial and agricultural activities associated with poor eating habits, and the unfavorable quality of the environment in which the population live. It is very difficult to assess the impact of individual factors associated with the distribution of breast and prostate cancers in Slovakia, as these are multifactorial diseases. The results of this study may have potential applications, such as being part of annual reports, and thus helping the involved agencies adopt administrative regulations, as well as make decisions on cancer control and prevention.

## 5. Conclusions

The death rate of cancer in the Slovak population is influenced by several factors. Particularly in doctors’ view, they are multifaceted diseases, and it is difficult to clearly identify the factors linked directly to the inception of the diseases. The most common factors are lifestyle, stress, diet, environmental quality, and others. The issue of cancer is very complex, and this article was not intended to deal with the issue in full, but only through the selected indicators. The submitted article addresses the issue of the assessment of standardized death rate of breast cancer in women and prostate cancer in men in Slovakia in the periods 2001–2006, 2007–2012, and 2013–2018. These results indicate that circulatory system diseases and cancer are dominant in the mortality structure of women but also in men. An increase in the mortality percentage due to cancer can be observed in both sexes. The population of Slovakia has undergone an overall dynamic and socially vast transformation since 1989, with implications for its current and future existence. One of the most significant changes is the shift in reproduction behavior, with the mortality course playing a significant role. In relation to the causes, it is clear that a more substantial approximation to Europe’s demographically mature populations is hindered, in particular, by adverse cardiovascular and cancer death rates [61]. Cancer has long been the second most common cause of death in Slovakia. In view of the spatial analysis of standardized death rate of breast and prostate cancer, it is possible to specify significantly different areas in Slovakia in terms of the achieved values. The results show a substantial degree of diversity between the Slovak districts. Slovakia is a country with major regional disparities resulting from historical, geographical, and cultural differences, but mainly from the different level of economic development and national composition stemming from differences in the social, educational, and overall economic level of regions. The result of these differences is fundamentally a different way and quality of life in the highly developed regions (localized mainly in the western part of Slovakia), such as Bratislava, Nitra, Trenčín, and Trnava. It is in these areas where the availability of medical care is conducted in well-equipped specialized oncology health care facilities. In underdeveloped regions of Southern and Eastern Slovakia, with the exception of the regional capitals of Košice and Prešov, the situation is completely different. The analyses confirm a significant spatial aspect of the distribution of the monitored indicator, which can also be observed at the regional level. This fact is also reflected in the specifications of considerable differences in a number of areas, such as the social and economic perspective, in particular in the east and the west. Identifying regions on the basis of the selected indicator of cancer and its values is important in view of the creation of measures in these problem regions. These measures must, in the first place, seek the optimization of lifestyles, especially activities which would discourage people from smoking and alcohol consumption, combat obesity, and promote physical activities, healthier diets, lifestyle change, protection from sunlight, and the like. From the analysis of the values and trends of incidence and mortality in Slovakia, there is a need to intensify prevention. In this context, we therefore consider it necessary to emphasize the very important role of the National Oncology Programme. Slovakia is the last EU country to have state-controlled screening. The invitation to a preventive medical examination will allow us to detect the three most common cancers in Slovakia—breast, cervix, and colon cancer. Our findings have revealed large disparities in the geographical distribution of breast cancer and prostate cancer. This geographical diversity thus helps to better target breast- and prostate-cancer determinants in Slovakia.
